# Cytotoxic Activity of Dendritic Cells as a Possible Mechanism of Negative Regulation of T Lymphocytes in Pulmonary Tuberculosis

**DOI:** 10.1155/2012/628635

**Published:** 2012-09-29

**Authors:** Ludmila V. Sakhno, Marina A. Tikhonova, Tamara V. Tyrinova, Olga Yu. Leplina, Ekaterina Ya. Shevela, Sergey D. Nikonov, Oleg A. Zhdanov, Alexander A. Ostanin, Elena R. Chernykh

**Affiliations:** ^1^Research Institute of Clinical Immunology, Russian Academy of Medical Sciences (RAMS), Siberian Branch (SB), Novosibirsk 630099, Russia; ^2^Novosibirsk Tuberculosis Clinical Hospital No. 1, Novosibirsk 630099, Russia

## Abstract

The PD-1/B7-H1-mediated induction of T cell apoptosis/anergy as a possible mechanism of immune response failure was studied in 76 patients with pulmonary tuberculosis (TB) with normal and low-proliferative response to antigens of *M. tuberculosis* (purified protein derivative (PPD)). It was revealed that dendritic cells (DCs), generated *in vitro* from patient blood monocytes with GM-CSF + IFN-**α**, were characterized by increased B7-H1 expression, upproduction of IL-10, and reducing of allostimulatory activity in mixed lymphocyte culture (MLC). Moreover, DCs of patients with TB were able to enhance T cell apoptosis and to block T-cell division in MLC. It was shown that neutralizing anti-PD1 antibodies significantly decreased the proapoptogenic/tolerogenic effect of DCs. Correlation analysis revealed a direct relationship between IL-10 production and level of B7-H1 expression in the general group of investigated patients. It was demonstrated that generation of healthy donor DCs in the presence of IL-10 led to an increase in the number of DCs-expressed B7-H1 molecule, DC proapoptogenic activity, and a decrease in their allostimulatory activity. Obviously, the revealed phenomenon of the PD-1/B7-H1-mediated pro-apoptogenic activity of DCs is clinically significant since the cytotoxic/tolerogenic potential of DCs is more pronounced in patients with PPD anergy.

## 1. Introduction

Recent studies have shown that dendritic cells (DCs) as innate immunity cells have cytotoxic functions and can suppress tumor cell growth [1–3]. This activity of DCs is mediated through expression of TNF family pro-apoptogenic molecules [1–3] and is enhanced in response to interferons [4, 5]. It was also shown that DCs are capable of expressing coinhibitory molecule B7-H1, which is a ligand for receptor-programmed cell death 1 (PD-1) [6, 7]. The fact that T cells activated through CD4^+^ and CD8^+^ T cell receptors express PD-1 enables the assumption that PD-1/PD-L1 (B7-H1) interactions between DCs and T lymphocytes could determine DCs' cytotoxic effect against T lymphocytes.

Negative regulation of T-lymphocytes through activation of the PD-1/PD-L1 (B7-H1) signaling pathway has been discussed in many immunopathological conditions, such as tumor growth, autoimmune diseases, and bacterial, viral, and parasitic infections [6, 8–10]. Tumor cells [6], lung and stomach epithelial cells [11, 12], monocytes [13, 14], and macrophages [15] may act as sources of increased B7-H1 expression. The role of DCs in exploiting the PD-1/PD-L1 (B7-H1) signaling pathway remains less studied. Increased B7-H1 expression on DCs as a possible mechanism of developing T cell dysfunctions has been discussed in human chronic viral hepatitis B [16] and has been demonstrated in SIV-infected primates [17].

According to literature data and from our research as well, increased apoptosis and anergy of T cells are characteristic attributes of tuberculosis infection, especially in the patients with decreased proliferative response to tuberculin-purified protein derivative (PPD) [18–20]. Our previous studies have also shown that monocytes and generated *in vitro* DCs from pulmonary tuberculosis (TB) patients are remarkable for increased interleukin-10 production [21]. Taking into consideration published data about IL-10-stimulating effect on B7-H1 expression [6], we assumed that involvement of the PD-1/B7-H1 signaling pathway in interaction between patient DCs and T lymphocytes may induce T-cell apoptosis/anergy and cause suppression of antigen-specific immune response in tuberculosis infection.

To verify this assumption, experiments were performed in order to compare B7-H1 expression in IFN-*α*-induced dendritic cells from donors and TB patients, to investigate the correlation between B7-H1 expression and IL-10 endogenous production level, and to evaluate DCs capability to induce T-cell apoptosis/anergy, as well as the role of the PD-1/B7-H1 signaling pathway in negative regulation of T-lymphocytes.

## 2. Materials and Methods

### 2.1. Patients

 The patients with active pulmonary tuberculosis were recruited from Novosibirsk Tuberculosis Clinical Hospital No. 1. The study included 76 pulmonary tuberculosis patients (47 men and 29 women between 16 and 63 years), of whom 28 patients had fibrous and cavernous TB, 38 patients had infiltrative TB, and 10 patients had disseminated TB. Positive sputum for mycobacteria tuberculosis (Mtb^+^) was detected in 46 patients; drug resistance was observed in 16 patients. TB patients were treated with standard antimicrobial therapy, including in case of first-line therapy the combination of tubazid, rifampicin, streptomycin, ethambutol and pyrazinamide, and as second line (in patients with multidrug resistance)—a combination of fluoroquinolone with amikacin or kanamycin, capriomycin, cycloserine, and PAS. The patients were examined after obtaining their informed consent. The control group consisted of 30 healthy blood donors comparable by sex and age.

### 2.2. Isolation of Cells and Evaluation of Proliferative Response

 Mononuclear cells (MNCs) were isolated from heparinized venous blood by Ficoll-Verographin density-gradient centrifugation, and cultivated in 96-well plates (0.1 × 10^6^ per well) in RPMI-1640 (Sigma-Aldrich, USA) medium, completed with 0.3 mg/mL L-glutamine, 5 mM HEPES buffer, 100 *μ*g/mL gentamycin, and 10% inactivated human AB serum. In order to stimulate cell-proliferative response, tuberculin-purified protein derivative (PPD) was used in a dose of 50 *μ*g/mL. Proliferation intensity was evaluated on the 6th day based on ^3^H thymidine incorporation (1 *μ*Ci per well), added 18 hours before the end of cultivation. Depending on proliferative response level (borderline value—12,500 cpm—represented the low value of interquartile range of healthy donor MNC PPD response), patients were divided into 2 subgroups: those with normal (>12,500 cpm; *n* = 50; subgroup 1) and reduced (<12,500 cpm; *n* = 26; subgroup 2) response to PPD.

### 2.3. Generation of Dendritic Cells

 Monocytes were isolated in 6-well plates (Nuclon, Denmark) by adhesion of MNC (3 × 10^6^ cells/mL) to the plastic in the presence of 5% human AB serum. DCs were generated from monocytes over 4 days in RPMI-1640 medium with 5% fetal calf serum (Biolot, St. Petersburg) in the presence of GM-CSF (40 ng/mL, Sigma-Aldrich) and IFN-*α* (1,000 U/mL, Roferon-A, Roche, Switzerland), followed by maturation over 24 hours in the presence of 10 *μ*g/mL lipopolysaccharide (LPS *E. coli* 0111:B4, Sigma-Aldrich). In the series of experiments, interleukin 10 (Sigma, 5 ng/mL) was added simultaneously with LPS. Evaluation of B7-H1 expression on DCs was conducted with phycoerythrin (PE)-labeled monoclonal anti-B7-H1 antibodies (Pharmingen, USA) using flow cytofluorometry (FASC Calibur, Becton Dickinson, USA). IL-10 concentration was determined in cultural supernatants of generated DCs using immune-enzyme assay kits according to the manufacturer's instructions (Vector-Best, Novosibirsk).

### 2.4. Evaluation of Allostimulatory Activity of DCs

 Allostimulatory activity of DCs was evaluated in mixed lymphocyte culture (MLC) after cultivation of donor MNC (0.1 × 10^6^/well) in round-bottom 96-well plates in the presence of allogenic DCs from healthy donors or TB patients in the ratio 10 : 1. Proliferation intensity was evaluated using radiometry on the 5th day based on ^3^H thymidine incorporation. The DC influence index (II_DC_) in MLC was calculated as the ratio of MNC-proliferative response in the presence of DCs to spontaneous MNC proliferation. In an additional series of experiments, the level of T-cell apoptosis in 3-day allo-MLC and proliferative response in 5-day allo-MLC were evaluated in the presence (experiment) and in the absence (control) of neutralizing antibodies against PD-1 (5 *μ*g/mL, J116; eBioscience; Functional Grade Purified). To evaluate the effect of anti-PD-1 antibodies on proliferative response level in MLC, the influence index (II_anti-PD1_) was calculated by the formula: II_anti-PD1_ = cpm experiment/cpm control.

### 2.5. Evaluation of PD-1 Expression and T-Lymphocyte Apoptosis/Proliferation Level

 PD-1 expression, apoptosis, and cell cycle analysis of T-lymphocytes were evaluated in 3-day allo-MLC (as described above). To evaluate PD-1 expression on CD4^+^ and CD8^+^ T-lymphocytes, MNCs (either freshly-isolated or stimulated in allo-MLC) were incubated with the following antibodies: FITC-labeled anti-CD3, PE-labeled anti-CD4 and anti-CD8 (Sorbent, Moscow), and APC-labeled anti-PD-1 (Becton Dickinson). The cell cycle and apoptosis level of CD4^+^ and CD8^+^ T lymphocytes were evaluated by three-color flow cytometry employing 7-amino actinomycin D (7-AAD, Calbiochem, Germany). Cell cycle analysis was conducted by evaluating DNA histograms (red fluorescence). The relative content of cells with diploid (cells in G_0_/G_1_ phases of the cell cycle) and hyperdiploid (cells in S, G_2_/M phases of the cell cycle) DNA sets was determined in gates of CD3^+^CD4^+^ or CD3^+^CD8^+^ T lymphocytes (green and orange fluorescence). Apoptotic cells with fragmentated DNA formed a characteristic hypodiploid peak. The results were expressed as a percent ratio of positive cells to the total quantity of CD3^+^CD4^+^ and CD3^+^CD8^+^ T lymphocytes (10,000 or more).

### 2.6. Statistical Analysis

 Statistical analysis was conducted using the software suite “Statistica 6.0.” To reveal significant differences between the parameters compared, the distribution-free Mann-Whitney *U*-criterion was employed. Differences were considered significant at the level of *P* < 0.05. To analyze correlation relationships between characteristics, the Spearman rank correlation coefficient was employed.

## 3. Results

### 3.1. TB Patients Have an Increased Number of B7-H1-Expressing IFN-DCs, and Higher IL-10 Production by DC Compared to Healthy Donors

**Figure 1:**
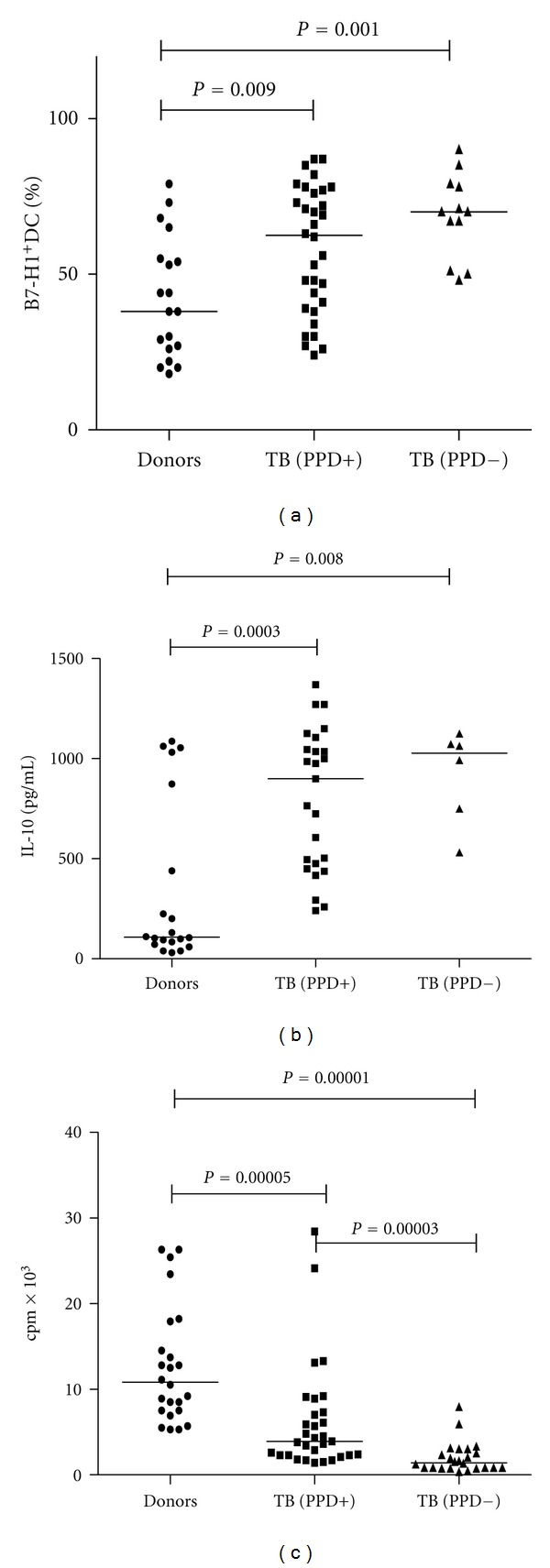
Expression of B7-H1 and production of IL-10 by patient DCs was higher, but their allostimulatory activity was lower than that of healthy donor DCs. (a) The expression of B7-H1 on the DCs in healthy donors (*n* = 19), PPD-reactive TB patients (PPD+; *n* = 32), and PPD-anergic TB patients (PPD−; *n* = 12) was detected by flow cytometry using anti-B7-H1 monoclonal antibodies. (b) The production of IL-10 by DCs was determined by ELISA in healthy donors (*n* = 20), PPD-reactive (*n* = 25) and PPD-anergic TB patients (*n* = 6). (c) The allostimulatory activity of DCs in MLC was detected in healthy donors (*n* = 24), PPD-reactive (*n* = 32), and PPD-anergic TB patients (*n* = 24). The median value was presented for each investigated group.

 Comparative study of B7-H1 expression on IFN-DCs from healthy donors and TB patients revealed that patient DC cultures contained significantly higher number of B7-H1^+^ DCs (61.0 ± 2.9 versus 42.3 ± 4.4%, resp.; *P*_*u*_ < 0.05). Increased level of B7-H1^+^ DCs was seen in both PPD-reactive and PPD-anergic patients (see Figure 1(a)). PPD-anergic patients, furthermore, were characterized by more pronounced enlargement of B7-H1^+^ DC contents. Also, TB patient DCs had higher IL-10 production (Figure 1(b)), and its level being the highest in DC cultures from PPD-anergic patients. Correlation analysis revealed a direct relationship between IL-10 production and level of B7-H1 expression both in the general group of investigated patients (*r* = 0.51;  *P* = 0.0004) and in the healthy donor group (*r* = 0.82;  *P* = 0.0005). Such strong correlation evidently indicates participation of IL-10 in autocrine regulation of B7-H1 molecule expression on DCs.

Enhanced B7-H1 expression coupled with elevated IL-10 production in patients was associated with impaired capability of TB patient DCs to stimulate T-cell proliferation in response to alloantigens in MLC (4,345 ± 709 versus 12,113 ± 1, 263 cpm in donors, *P*_*u*_ < 0.05). Decrease of DCs allostimulatory activity was revealed in both PPD reactive and PPD-anergic patients; in the latter, though, it was significantly more pronounced (Figure 1(c)). Thus, the proliferation level and MLC stimulation index in PPD-anergic patients were significantly lower than in PPD-reactive patients.

### 3.2. The Expression of PD-1 on T Cells Is Amplified in MLC When Stimulated DCs from Healthy Donors and Patients with TB

The decrease in DCs allostimulatory activity might be related to induction of T-cell apoptosis/anergy as a result of recruitment of the PD-1/PD-L1 (B7-H1) signaling pathway. Actually, T-cell activation is shown to be accompanied by increasing PD-1 expression, and there are some data indicating a direct relationship between B7-H1 expression on antigen-presenting cells and T-lymphocyte PD-1 receptor expression [17]. Therefore, we questioned whether patient DCs are more capable of stimulating PD-1 expression when T-cells are activated in MLC. Both donor and patient DCs stimulated PD-1 expression in CD4^+^ and CD8^+^ T cells. Contrary to our expectations, DCs from healthy donors and TB patients do not significantly differ in stimulating activity on PD-1 expression (Table 1).

Nonetheless, DC-induced enhancement of PD-1 expression on T-cells in MLC we discovered confirms the possibility of engagement of PD-1/PD-L1 (B7-H1) pathway during DC-T cell interaction.

### 3.3. DCs from TB Patients Exhibit Increased Apoptogenic/Tolerogenic Activity in Allo-MLC

**Figure 2:**
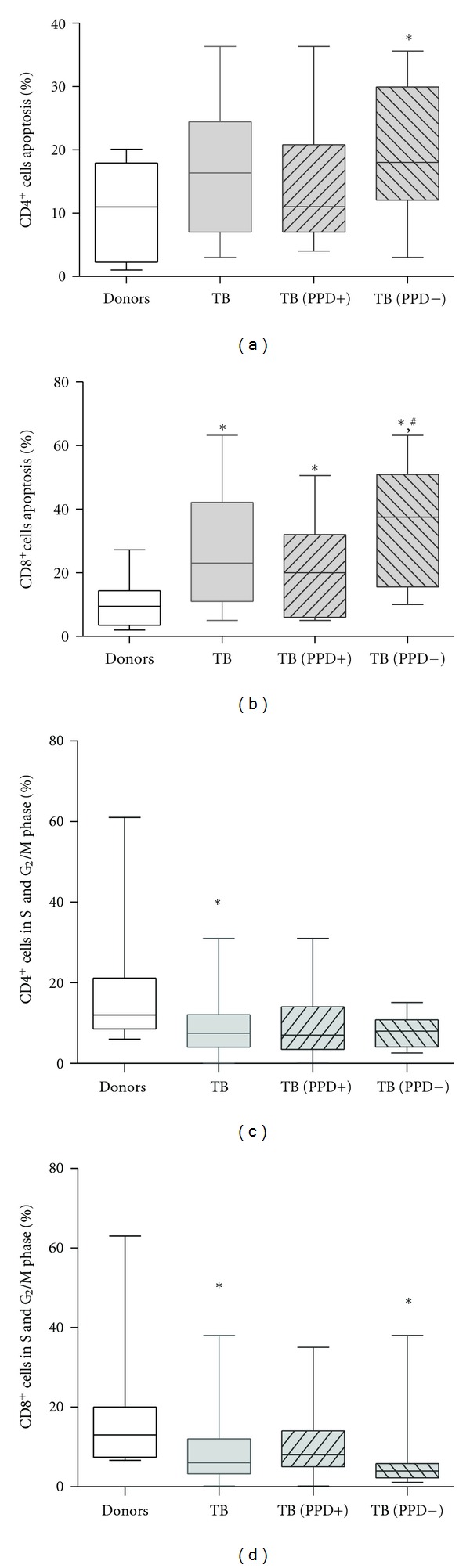
The apoptosis of T cells in allo-MLC stimulated with TB patient DCs was higher, and T cell proliferation was lower than that stimulated with healthy donor DCs. Date are presented in median value and whiskers (min to max). (a) After 3-day MLC stimulated with DCs of (PPD-) TB patients, the number of apoptotic CD4^+^ cells was significantly higher compared with MLC induced by healthy donor DCs. (b) After 3-day MLC stimulated with TB patient DCs the number of apoptotic CD8^+^ cells was significantly higher than that in MLC stimulated with healthy donor DCs. The number of apoptotic CD8^+^ cells in MLC stimulated with DCs of PPD-anergic TB patients was significantly higher than that in MLC cultures with healthy donor DCs (*P < 0.05) or PPD-responsive patient DCs (^#^ *P* < 0.05). (c) The number of CD4^+^ cells in S and G_2_/M phase of cell cycle in allo-MLC stimulated with TB patient DCs was higher than that stimulated with healthy donor DCs. (d) The number of CD8^+^ cells in S and G_2_/M phase of cell cycle in allo-MLC stimulated with patient DCs and DCs of PPD-anergic patients was higher than that stimulated with healthy donor DCs.

 Increased B7-H1 expression on DCs from TB patients enabled the assumption that reduced T-cell proliferation in MLC may be caused by the DC higher potential to induce apoptosis/anergy of responding T cells through the B7-H1 and PD-1 interaction. In order to test this hypothesis, the apoptosis level and number of T-lymphocytes in S, G_2_/M phases of the cell cycle were examined in MLC induced with donor and patient DCs. In healthy donors the level of apoptotic CD4^+^ and CD8^+^ T-cells among freshly isolated MNC did not exceed 3%. T-cell activation in MLC induced by donor DCs was accompanied by a 3-fold increase in apoptotic CD4^+^ and CD8^+^ T-lymphocytes (Figures 2(a) and 2(b)). At the same time, cocultivation of T-cells with TB patient DCs leads to more pronounced increase in the number of apoptotic T lymphocytes, statistically significant in the CD3^+^CD8^+^ T-cell population (Figures 2(a) and 2(b)). Of interest, CD3^+^CD8^+^ T-cells had greater sensitivity to apoptosis-inducing activity of patient DCs than CD3^+^CD4^+^ T-cells (26.9 ± 3.0% versus 17.0 ± 2.0%, resp.; *P* = 0.007). It is also noteworthy that DCs from PPD-anergic patients had more pronounced pro-apoptogenic activity as compared with the opposite group.

Cell cycle analysis showed that if MLC was induced with donor DCs, on average 13% of CD4^+^ and CD8^+^ T cells were in S, G_2_/M phases of the cell cycle. The fraction of proliferating CD4^+^ and CD8^+^ T-cells in MLC induced with TB patient DCs was significantly lower (Figures 2(c) and 2(d)). Moreover, the minimal number of cycling T-cells was observed in MLC stimulated with DCs from PPD-anergic patients. Correspondently, the ratio of apoptotic to proliferating T cells in MLC stimulated with patient DCs was on average 3–6 times higher than in MLC induced by donor DCs. Thus, the response of T-cells stimulated with TB patient DCs (with an increased B7-H1 expression level) was characterized by a higher apoptosis level and less-intensive proliferation, compared to the response induced with donor DCs. 

### 3.4. The Number of PD-1^+^CD4^+^ and PD-1^+^CD8^+^ T Lymphocytes in Peripheral Blood of PPD-Anergic TB Patients Is Elevated as Compared to Healthy Donors

**Figure 3:**
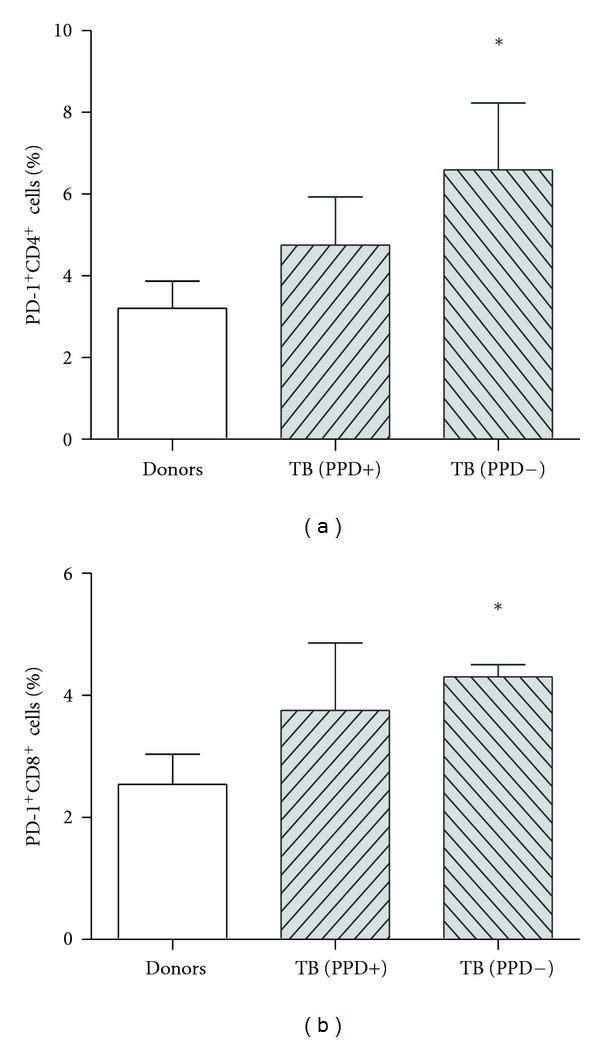
The number of freshly isolated PD-1^+^CD4^+^ and PD-1^+^CD8^+^ cells in PPD-anergic TB patients was higher than that of healthy donors. The numbers of PD-1^+^CD4^+^ (M ± S.E.) cells (a) and of PD-1^+^CD8^+^ cells (b) were detected in the peripheral blood of healthy donors: PPD-reactive and PPD-anergic TB patients.

 Despite the similar capability of patient and donor DCs to stimulate PD-1 expression on T-cells, freshly isolated MNCs of PPD anergic patients differed by significantly higher number of PD-1^+^CD4^+^ and PD-1^+^CD8^+^ T-lymphocytes. At the same time enhanced level of PD-1 positive CD4^+^ and CD8^+^ T-cells in PPD-reactive patients was displayed as a trend (Figure 3). 

Therefore, suppression of antigen-specific response in tuberculin-anergic TB patients might be conditioned not only by increased B7-H1 expression on DCs, but also by a higher PD-1 level on T-lymphocytes.

### 3.5. Blocking Anti-PD-1 Antibodies Decreases T-Lymphocyte Apoptosis Level in Allo-MLC

**Figure 4:**
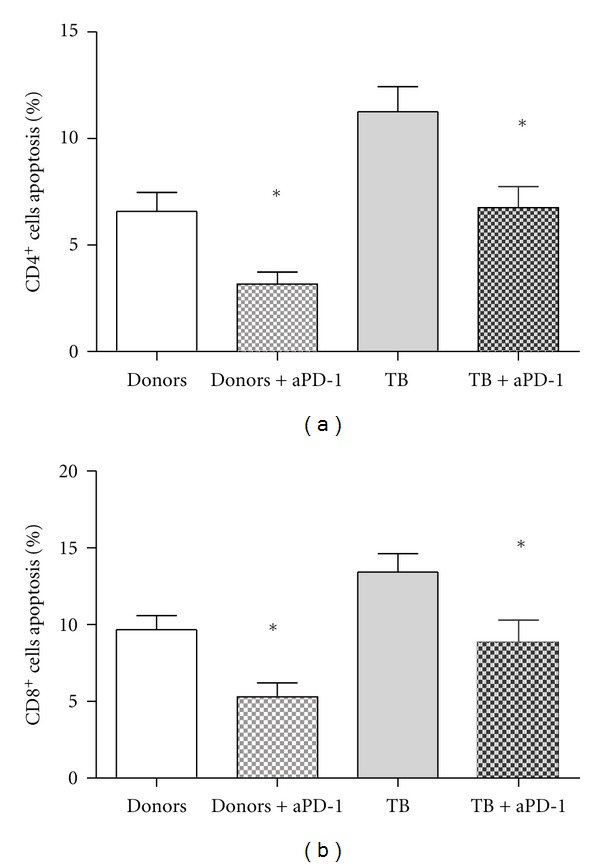
Addition of neutralizing anti-PD-1 antibodies in 3-day MLC stimulated with healthy donor and TB patient DCs was accompanied by decrease in CD3^+^CD4^+^ and CD3^+^CD8^+^ T-cell apoptosis. The number of apoptotic CD3^+^CD4^+^ (M ± S.E.) (a) and CD3^+^CD8^+^ (b) T-cells was determined in allo-MLC stimulated with healthy donor and TB patient DCs in the absence and presence of anti-PD-1 antibodies (5 *μ*g/mL).

 To demonstrate that T-cell apoptosis is directly mediated through the involvement of PD-1/B7-H1 signaling pathway during their interaction with DCs, a series of experiments with blocking anti-PD-1 antibodies has been performed (Figure 4). 

Addition of neutralizing anti-PD-1 antibodies in 3-day MLC stimulated with healthy donor DCs was accompanied by decrease in CD3^+^CD4^+^ and CD3^+^CD8^+^ T-cell apoptosis by 49% and 44%, respectively (Figures 4(a) and 4(b)). The same effect though slightly less marked (reduced on average by 35%) was demonstrated in MLC stimulated by TB patient DCs (Figures 4(a) and 4(b)).

### 3.6. Blocking Effect of Anti-PD-1 Antibodies on the Proliferative Response of T-Cells Depends on B7-H1 Expression on TB Patient DCs

 Evaluation of T-cell proliferative activity in MLC showed that addition of anti-PD-1 antibodies to MLC stimulated with TB patient DCs had no effect on the proliferative response of donor T-cells at the mean values. Nevertheless, we revealed a direct relationship between the influence index of neutralizing antibodies and the B7-H1 expression level on patient DCs (*r* = 0.65;  *P* < 0.05;  *n* = 10). Thus, if *in vitro* generated patient DCs expressed B7-H1 above the upper quartile of the normal range (>67%; an average of 81 ± 6%), the addition of anti-PD-1 antibodies was accompanied by a 2-fold increasing in proliferation of responding T-cells (II_anti-PD1_ = 1.8 ± 0.2 units; *p*_*u*_ = 0.018). In turn, when the number of generated B7-H1^+^ DCs was significantly lower (averaged 49 ± 5%), neutralizing antibodies had no effect on T-cell proliferation in MLC (II_anti-PD1_ = 0.92 ± 0.06 units).

### 3.7. Exogenous IL-10 Increases B7-H1 Expression, IL-10 Production, and Pro-Apoptogenic Activity, but Reduces Allostimulatory Activity of Donor DCs

 The direct correlation between B7-H1 expression and IL-10 production by DCs enabled us to suggest the important role of endogenous IL-10 in regulation of B7-H1 expression. To check this possibility, we studied the effect of exogenous IL-10 added to donor DCs on B7-H1 expression and allostimulatory activity of DCs. As shown in Table 2, IL-10 increased the number of B7-H1 positive DCs and decreased their allostimulatory activity. 

Moreover, addition of IL-10 to DC cultures enhanced their pro-apoptogenic activity towards the CD3^+^CD8^+^ T-lymphocytes (Table 2). At the same time, there were no differences in number of proliferating T-cells (in S, G_2_/M phases of the cell cycle) in MLC stimulated by both intact and IL-10 pretreated DCs (Table 2). Therefore, exogenous IL-10 increased the pro-apoptogenic activity of DCs, but did not influence on their capacity to block T-cell cycle progression.

## 4. Discussion

Recent data on the ability of DCs to express B7-H1 suggested that activation of PD-1/PD-L1 (B7-H1) signaling pathway upon DCs and T-lymphocyte interaction could mediate DC-cytotoxic activity against activated T-lymphocytes and increased expression of B7-H1 on DCs in tumor growth [6] and virus infections, and in such a way result in T cell deficiency [16, 17]. Considering also the fact that increased T-cell apoptosis/anergy in TB patients is associated with pronounced changes in the functional activity of monocytes and generated *in vitro *DCs [21], we have suggested that T-cell dysfunction in tuberculosis infection could be also conditioned by enhanced DC cytotoxic activity mediated through PD-1/PD-L1 (B7-H1) signaling pathway. 

To check this idea we evaluated the expression of B7-H1^+^ on generated *in vitro* IFN-*α*-DCs and for the first the time showed that DC cultures of TB patients contained an increased number of B7-H1^+^ cells. Since DCs of TB patients were also characterized by higher production of IL-10, we assumed that increased expression of B7-H1^+^ on DCs may be caused by enhanced production of IL-10. Actually, IL-10 was shown to increase B7-H1 expression on myeloid DCs generated in the presence of IL-4 [7]. In this aspect our data about direct correlation between IL-10 production and B7-H1 expression support this possibility. 

There is evidence that B7-H1 expression on myeloid DCs is also considerably increased in the presence of IFN-*α* and IFN-*γ* [16], and IFN-*α*-DCs are characterized by significantly higher B7-H1 expression than DCs obtained in the standard protocol with IL-4 [22]. However, differences in B7-H1 expression between donor and patient IFN-*α*-DCs as well as lower production of IFN-*α* and IFN-IF-*γ* by patient DCs (data not shown) skew to the mind that elevated count of B7-H1^+^ DCs in TB patients is not attributed to the effect of interferon. 

We also found that elevated expression of B7-H1 on patient DCs was associated with their reduced capacity to stimulate proliferation of T cells in MLC that is consistent with the previous reports on the decreased allostimulatory activity of TB patient DCs generated in the presence of IL-4 [23, 24]. Moreover, we showed that lower T cell proliferation was associated with both blocking of T-cell proliferation and a higher level of T cell apoptosis as well. Of note, we revealed the different sensitivity of T-cells to DC-mediated apoptosis. In particular, CD8^+^ T-lymphocytes were found to be more susceptible to apoptosis than CD4^+^ T-cells. This supports the assumption that DCs could play an important role in negative regulation of CD8^+^ cytotoxic T-lymphocytes in tuberculosis infection. Since donor and patient DCs did not differ in their ability to induce PD-1 expression on CD4^+^ and CD8^+^ T-lymphocytes, the enhanced T-cell apoptosis in MLC induced with patient DCs is evidently related to an increased level of B7-H1 expression. Really, a pronounced decrease in the apoptotic cell number and increase in proliferating T-cells in MLC which we observed in the presence of neutralizing anti-PD-1 antibodies (which block the PD-1/B7-H1 signaling pathway) are direct arguments for engagement of the PD-1/B7-H1 pathway in pro-apoptogenic/tolerogenic activity of DCs. 

The results of our studies also have demonstrated that DCs in PPD-anergic patients with the higher B7-H1 expression are characterized by lower ability to stimulate T cell proliferation in MLC and higher capacity to induce T lymphocyte apoptosis, as compared to DCs in PPD-reactive patients. In addition, we showed for the first time that PPD-anergic patients are differed by significantly higher content of circulating PD-1^+^CD4^+^ and PD-1^+^CD8^+^ T lymphocytes, as compared to healthy donors. These data confirm the hypothesis that increased pro-apoptogenic and tolerogenic DC activity is not only *in vitro* phenomenon, but may be an important mechanism underlying *in vivo* impairment of the antigen-specific T-cell response in TB patients. 

Interestingly, anti-PD-1 antibodies cannot fully cancel T-cell apoptosis induced by donor and patient DCs. This indicates the possible engagement of other apoptotic mechanisms, which may be mediated, for example, by Fas-L, TRAIL, TNF-*α*, or perforin. Actually, the role of these molecules in initiating of programmed cell death was demonstrated in an investigation of cytotoxic activity of DCs against tumor line cells [25]. It is likely that such a data can also explain the different sensitivity of CD4^+^ and CD8^+^ T-cells to the pro-apoptogenic effect of patient DCs, despite a similar level of PD-1 expression on CD4^+^ and CD8^+^ T-lymphocytes.

To clarify the role of IL-10 in regulating B7-H1 expression, we also investigated the effect of exogenous IL-10 on expression of B7-H1 molecules and allostimulatory activity of donor DCs. The results obtained have shown that increased B7-H1 expression on IL-10 pretreated donor DCs was accompanied by decreased allostimulatory and enhanced pro-apoptogenic activity of DCs against CD3^+^CD8^+^ T lymphocytes. This is in agreement with our data on higher CD8^+^ T-lymphocyte sensitivity to the pro-apoptogenic effect of patient DCs. IL-10 inhibitory effect on DCs is a well-known fact [26, 27]. The results presented here indicate that along with direct suppressive activity of IL-10 on T-cell proliferation the triggering of PD-1/B7-H1 signaling pathway by stimulation of B7-H1 expression on DCs can be considered as another mechanism mediating IL-10 suppressive activity.

Taken together, our data support the hypothesis that DCs generated *in vitro* in the presence of IFN-*α* are capable of inducing apoptosis/anergy of T-cells through the PD-1/B7-H1 signaling pathway, and the increased B7-H1 expression on patient DCs could result in impairment of T cell antigen-specific response.

## Figures and Tables

**Table 1:** The numbers of PD-1^+^CD4^+^ and PD-1^+^CD8^+^ T cells among the healthy donor MNC.

	*N*	PD-1^+^CD4^+^ T-cells (%)	PD-1^+^CD8^+^ T-cells (%)
Freshly isolated MNC	7	3.2 ± 0.7	2.5 ± 0.5
MNC stimulated with healthy donor DCs	6	5.1 ± 0.9*	8.8 ± 1.2*
MNC stimulated with TB patient DCs	23	8.1 ± 0.9*	10.2 ± 0.9*

The numbers (%) of PD-1^+^CD4^+^ and PD-1^+^CD8^+^ T-cells in peripheral blood MNC of healthy donors and in MNC cultivated for 3 days with allogeneic DCs were presented. The percentage of PD-1^+^CD4^+^ and PD-1^+^CD8^+^ T-cells in the cultures of MNC stimulated with TB patient DCs or healthy donor DCs was statistically higher than the number of PD-1^+^CD4^+^ and PD-1^+^CD8^+^ T-cells in freshly isolated MNC separated from peripheral blood (*P_*u*_ < 0.05).

**Table 2:** The effects of exogenous IL-10 on phenotypic and functional properties of healthy donor DCs.

	*N*	LPS	LPS + IL-10	*P* value
B7-H1^+^DCs (%)	14	40.4 ± 4.1	48.1 ± 5.3	0.012
Allostimulatory activity in MLC (cpm)	14	13,432 ± 1,841	9,230 ± 992	0.011
Index of stimulation in MLC	14	35.6 ± 6.9	23.7 ± 3.8	0.012
Apoptosis of CD3^+^CD4^+^ cells in MLC (%)	14	8.3 ± 1.8	10.4 ± 2.3	0.2
Apoptosis of CD3^+^CD8^+^ cells in MLC (%)	14	11.2 ± 2.0	17.0 ± 2.8	0.006
CD3^+^CD4^+^ cells in S and G_2_/M phase (%)	14	17.9 ± 3.3	15.8 ± 3.2	0.18
CD3^+^CD8^+^ cells in S and G_2_/M phase (%)	14	18.6 ± 2.2	18.9 ± 2.5	0.92

The relative number of B7-H1^+^DCs (M ± S.E.) was higher in the culture of DCs generated in the presence of IL-10. The allostimulatory activity of DCs generated in the presence of IL-10 was lower than that of DCs generated without IL-10. The generation of healthy donor DCs in the presence of IL-10 was accompanied by increasing CD3^+^CD8^+^ cell apoptosis in MLC stimulated by those DCs.
